# Safety of Resuming Tumor Necrosis Factor Inhibitors in Ankylosing Spondylitis Patients Concomitant with the Treatment of Active Tuberculosis: A Retrospective Nationwide Registry of the Korean Society of Spondyloarthritis Research

**DOI:** 10.1371/journal.pone.0153816

**Published:** 2016-04-21

**Authors:** Hye Won Kim, Seong Ryul Kwon, Kyong-Hee Jung, Seong-Kyu Kim, Han Joo Baek, Mi Ryung Seo, So-Young Bang, Hye-Soon Lee, Chang-Hee Suh, Ju Yang Jung, Chang-Nam Son, Seung Cheol Shim, Sang-Hoon Lee, Seung-Geun Lee, Yeon-Ah Lee, Eun Young Lee, Tae-Hwan Kim, Yong-Gil Kim, Korean Society of Spondyloarthritis Research

**Affiliations:** 1 Department of Internal Medicine, Yonsei University College of Medicine, Division of Rheumatology, Gangnam Severance Hospital, Seoul, Korea; 2 Division of Rheumatology, Department of Internal Medicine, Inha University School of Medicine, Incheon, Korea; 3 Division of Rheumatology, Department of Internal Medicine, Arthritis and Autoimmunity Research Center, Catholic University of Daegu School of Medicine, Daegu, Korea; 4 Division of Rheumatology, Department of Internal Medicine, Gachon University Gil Hospital, Incheon, Korea; 5 Division of Rheumatology, Department of Internal Medicine, Hanyang University Guri Hospital, Guri, Korea; 6 Division of Rheumatology, Department of Internal Medicine, Ajou University Hospital, Suwon, Korea; 7 Division of Rheumatology, Department of Internal Medicine, Keimyung University Dongsan Medical Center, Daegu, Korea; 8 Division of Rheumatology, Department of Internal Medicine, Chungnam National University Hospital, Daejeon, Korea; 9 Department of Rheumatology, Center of Arthritis and Rheumatism, Kyung Hee University Hospital at Gangdong, Seoul, Korea; 10 Division of Rheumatology, Department of Internal Medicine, Pusan National University Hospital, Busan, Korea; 11 Division of Rheumatology, Department of Internal Medicine, Kyung Hee University Medical center, Seoul, Korea; 12 Division of Rheumatology, Department of Internal Medicine, Seoul National University Hospital, Seoul, Korea; 13 Division of Rheumatology, Hanyang University Hospital for Rheumatic Diseases, Seoul, Korea; 14 Division of Rheumatology, Department of Internal Medicine, University of Ulsan College of Medicine, Asan Medical Center, Seoul, Korea; Oregon Health & Science University, UNITED STATES

## Abstract

**Backgrounds:**

Patients who develop an active tuberculosis infection during tumor necrosis factor (TNF) inhibitor treatment typically discontinue TNF inhibitor and receive standard anti-tuberculosis treatment. However, there is currently insufficient information on patient outcomes following resumption of TNF inhibitor treatment during ongoing anti- tuberculosis treatment. Our study was designed to investigate the safety of resuming TNF inhibitors in ankylosing spondylitis (AS) patients who developed tuberculosis as a complication of the use of TNF inhibitors.

**Methods:**

Through the nationwide registry of the Korean Society of Spondyloarthritis Research, 3929 AS patients who were prescribed TNF inhibitors were recruited between June 2003 and June 2014 at fourteen referral hospitals. Clinical information was analyzed about the patients who experienced tuberculosis after exposure to TNF inhibitors. The clinical features of resumers and non-resumers of TNF inhibitors were compared and the outcomes of tuberculosis were surveyed individually.

**Findings:**

Fifty-six AS patients were treated for tuberculosis associated with TNF inhibitors. Among them, 23 patients resumed TNF inhibitors, and these patients were found to be exposed to TNF inhibitors for a longer period of time and experienced more frequent disease flare-up after discontinuation of TNF inhibitors compared with those who did not resume. Fifteen patients resumed TNF inhibitors during anti-tuberculosis treatment (early resumers) and 8 after completion of anti-tuberculosis treatment (late resumers). Median time to resuming TNF inhibitor from tuberculosis was 3.3 and 9.0 months in the early and late resumers, respectively. Tuberculosis was treated successfully in all resumers and did not relapse in any of them during follow-up (median 33.8 [IQR; 20.8–66.7] months).

**Conclusions:**

Instances of tuberculosis were treated successfully in our AS patients, even when given concomitantly with TNF inhibitors. We suggest that early resumption of TNF inhibitors in AS patients could be safe under effective coverage of tuberculosis.

## Introduction

Tuberculosis is a serious complication associated with tumor necrosis factor (TNF) blockade therapy [1–3]. Risk factors for tuberculosis in autoimmune disease include use of anti-TNF monoclonal antibody, old age, rheumatic disease itself, concomitant disease-modifying antirheumatic drugs (DMARDs), glucocorticosteroids and previous tuberculosis infection history [4–7]. Although AS patients tend to have fewer conventional tuberculosis-related comorbidities than rheumatoid arthritis (RA) patients, the growing use of TNF inhibitors to treat AS has raised incidences of tuberculosis to a level similar to that in RA patients [8]. According to the Korean data of Health Insurance Review and Assessment Service, the incidence of tuberculosis ensuing TNF inhibitors are reported to be 715/100,000 person-years for AS patients and 1143/100,000 person-years for RA patients [9]. Among 8421 cases of TNF inhibitor users, etanercept was given for 47% of the patients, infliximab for 23.9% and adalimumab for 29.1% of the patients. The incidence rate ratio when compared with the etanercept group was 6.80 for infliximab and 3.45 for adalimumab [9].

When active tuberculosis occurs in patients with AS on TNF inhibitors, a full course of anti-tuberculosis therapy and withdrawal of the biologics is generally recommended [5, 10, 11]. However, managing and preventing flares of AS following discontinuation of TNF inhibitor is as much of a challenge to the clinicians treating tuberculosis itself.

Although novel approaches are under investigation, treatment options are limited, particularly in non-steroidal anti-inflammatory drug (NSAID)-refractory AS [12]. The exception is the use of TNF inhibitors, which have had major successes in the clinical field. Among these successes are reducing axial and extra-articular disease activity, improving physical function, and quality of life with potential of disease modification [13, 14]. However, complete withdrawal of anti-TNF therapy or dose reduction leads to clinical relapse of the AS within several weeks to months [15, 16]. Meanwhile, the use of corticosteroid or low-risk biologics such as rituximab, tocilizumab, or abatacept may be considered for severe flares in RA patients being treated for active tuberculosis [17]. Therefore, to control underlying AS, we have to make the best of the armaments at our disposal. However, the safety and optimal timing for reinitiating TNF inhibitor in patients with active tuberculosis associated with previous or concurrent use of TNF inhibitors are unknown. Recommendations are conflicting between delaying and continuing TNF inhibitors on the back of tuberculosis treatment. The consensus of the Tuberculosis Network European Trialsgroup states delay for TNF inhibitor as long as possible in the absence of specific clinical trials. In contrast, the guidelines of the British Thoracic Society recommended continuation of a TNF inhibitor if clinical benefits of a TNF inhibitor is considered far outweigh the risks [5, 10]. Recently, individualized introduction of TNF inhibitors or DMARDs according to AS disease activity and risk of tuberculosis activation has been proposed in an expert opinion [17]. The inconsistencies and conflicts between recommendations, along with the clinician’s necessity to control AS disease activity, have led many physicians in our registry to individually resume TNF inhibitors in AS patients who have been diagnosed as having active tuberculosis.

Therefore, in order to critically evaluate strategy of resuming TNF inhibitors after tuberculosis treatment, we sought to assess the safety of resuming TNF inhibitors concomitantly with or after completing anti-tuberculosis chemotherapy based on a present nationwide study in an intermediate tuberculosis burden area.

## Methods

### Patients

**Fig 1:**
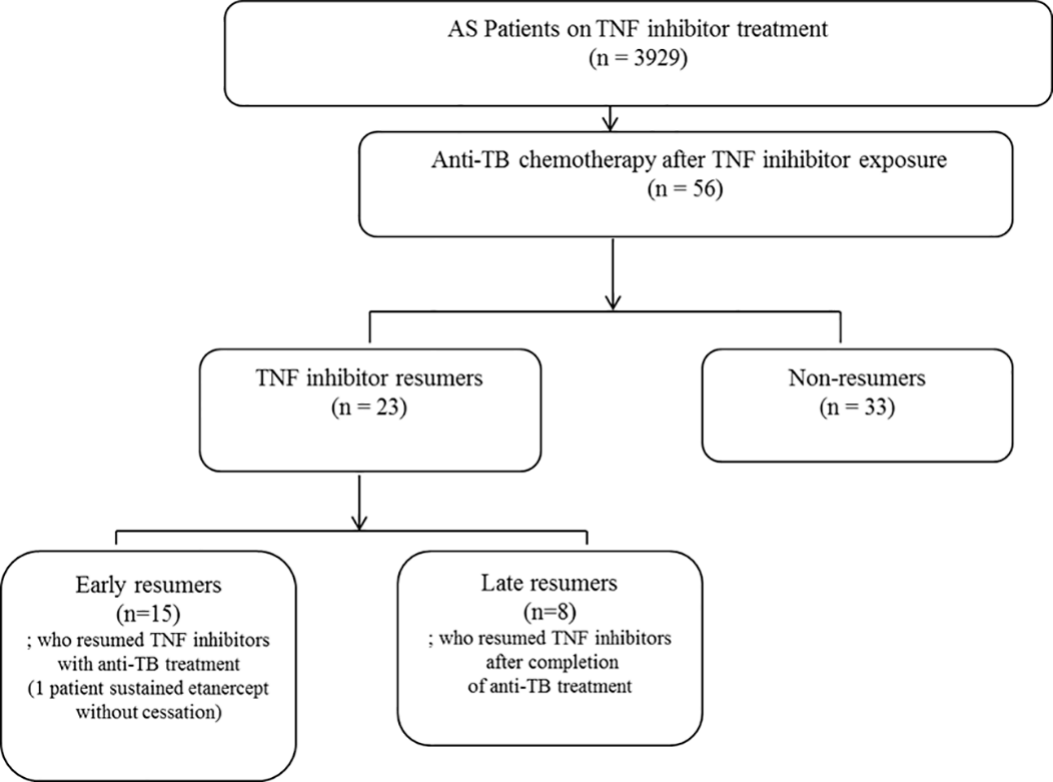
Flow diagram of patient selection. AS, ankylosing spondylitis; TB, tuberculosis; TNF, tumor necrosis factor.

AS patients who had been treated with TNF inhibitors in 14 referral hospitals between June 2003 and June 2014 were recruited in the registry of the Korean Society of Spondyloarthritis Research (KSSR). All patients met the 1984 modified New York criteria for AS [18]. The histories of previous tuberculosis and treatment of latent tuberculosis infection (LTBI) were collected, which is mandatorily ascertained before initiation of TNF inhibitors during clinical practice by Korean guidelines regarding tuberculosis. Briefly, either positive tuberculosis skin test or QuantiFERON releasing assay without evidence of active tuberculosis would be regarded as having LTBI and treated with appropriate regimen (9 months of isoniazid, 4 months of rifampin or 3 months of isoniazid plus rifampin) [19]. Patients who developed active tuberculosis after initiating TNF inhibitors were identified and their clinical information was collected from the registry database. The patients who developed tuberculosis after 1-yr discontinuation of TNF inhibitors were excluded. We specifically focused on the recruitment of those who resumed TNF inhibitors after tuberculosis associated with recent or current TNF inhibitor therapy. The outcome of tuberculosis was surveyed in these patients who resumed TNF inhibitors. Fig 1 shows the flow of patient enrollments.

This multi-site study protocol was approved by the institutional review boards from all participating sites including Eulji General Hospital, Inha University School of Medicine, Catholic University of Daegu School of Medicine, Gachon University Gil Hospital, Hanyang University Guri Hospital, Ajou University Hospital, Keimyung University Dongsan Medical Center, Chungnam National University Hospital, Kyung Hee University Hospital at Gangdong, Pusan National University Hospital, Kyung Hee University Medical center, Seoul National University Hospital, Hanyang University Hospital for Rheumatic Disease and Asan Medical Center. Each review boards waived the requirement for the investigator to obtain a signed consent form for all subjects as the research presents no more than minimal risk of harm to subjects. The review boards found that the only record linking the subject and the research would be the consent document and the waiver or alteration of normal consent procedures will not adversely affect the rights and welfare of the subjects. All patient records/information were anonymized.

### Diagnosis of tuberculosis infection

Active tuberculosis was diagnosed based on microbiological or pathological evidence of *Mycobacterium tuberculosis* infection; a positive acid-fast bacillus (AFB) culture, a positive *Mycobacterium tuberculosis* polymerase chain reaction, and the presence of caseating granulomas on tissue specimens. Patients were also diagnosed as having active tuberculosis if high clinical suspicion was supported by radiographic evidence or high adenosine deaminase levels in adequate specimens together with clinical improvement upon anti-tuberculosis treatment.

In cases in which a patient was treated with two or more TNF inhibitors, the last TNF inhibitor used before the onset of tuberculosis was regarded as the causative agent. Follow-up was censored at the most recent follow-up or death, whichever came first.

### Resuming TNF inhibitors

TNF inhibitor treatment was stopped if active tuberculosis diagnosed unless a therapeutic benefit from continuation of TNF inhibitor treatment was expected. All patients were treated with adequate anti-tuberculosis treatment. Briefly, first-line agents (isoniazid, rifampin, pyrazinamide, and ethambutol) were applied for 6 months in pulmonary tuberculosis and for 9–12 months in extrapulmonary tuberculosis. TNF inhibitors were resumed at physician’s discretion, mostly when clinically indicated in patients experiencing a flare-up of AS activity with worsened pain or immobility, usually after checking anti-tuberculosis drug sensitivity. Researchers reported the AS disease activity at the close time after suspension of TNF inhibitor as being improved, stable or being worsened grounded on patients’ medical records. Selection of a specific TNF inhibitor was at the discretion of each attending physician. To detect a recurrence of tuberculosis, our patients were observed for active tuberculosis infection during routine clinical practice if they contacted patients with active tuberculosis or showed symptoms and signs suspicious of active tuberculosis. The outcome of tuberculosis was surveyed in the patients who resumed TNF inhibitors if they failed initial tuberculosis treatment or had been hospitalized.

The characteristics of resumers and non-resumers of TNF inhibitors after initiation of anti-tuberculosis treatment were compared. Resumers were further separated into two subgroups: early resumers (who resumed TNF inhibitors during anti-tuberculosis treatment) and late resumers (who resumed TNF inhibitor after completion of anti-tuberculosis treatment).

### Statistical analysis

Data are expressed as medians (IQR; interquartile ranges), and differences were analyzed using the Chi-squared test and Student’s t-test or the Mann-Whitney U test (for continuous variables) as appropriate. All statistical analyses were performed using SPSS 19.0 for Windows (IBM SPSS Inc., Chicago, IL).

## Results

### Clinical features of patients with tuberculosis associated with TNF inhibitor

As shown in Table 1, a total of 3929 AS patients who were treated with TNF inhibitors were identified. Among them, 56 patients (median 43.0 years, male 89.3%, and median disease duration of 8.9 years) were diagnosed as tuberculosis. Median exposure duration of TNF inhibitor was 27.8 (IQR 11.2–64.0) months. Anti-TNF agents used at the time of diagnosis of tuberculosis were infliximab (n = 23, 41.7%), adalimumab (n = 21, 37.5%), etanercept (n = 12, 21.4%) in order of frequency.

**Table 1:** Comparison of clinical features between resumers and non-resumers of TNF inhibitors in AS patients who experienced active tuberculosis infection.

	Total patients (n = 56)	Resumers (n = 23)	Non-resumers (n = 33)	*P*
Age, yr (median, IQR)	43.0 (32.9–55.8)	43.3 (32.8–47.7)	39.3 (33.3–61.9)	0.16
Male sex, n (%)	50 (89.3)	22 (95.7)	28 (84.8)	0.19
Disease duration, yr (median, IQR)	8.9 (5.9–13.4)	9.3 (6.1–13.4)	8.5 (5.9–13.9)	0.54
BMI, kg/m^2^ (median, IQR)	23.5 (20.3–25.0)	24.0 (22.0–26.1)	22.2 (19.7–24.8)	0.18
LTBI, n (%)	15 (26.8)	6 (26.0)	9 (27.3)	0.94
Previous tuberculosis history, n (%)	10 (17.8)	5 (21.7)	5 (15.2)	0.53
Concomitant treatment				
Methotrexate, n (%)	18 (32.1)	7 (30.4)	11 (33.3)	0.82
Sulfasalazine, n (%)	8 (14.3)	2 (8.7)	6 (18.2)	0.32
Steroids, n (%)	11 (19.6)	4 (17.4)	7 (21.2)	0.72
Type of TNFi at tuberculosis infection				0.07
Infliximab, n (%)	23 (41.7)	13 (56.5)	10 (30.3)	
Adalimumab, n (%)	21 (37.5)	8 (34.8)	13 (39.4)	
Etanercept, n (%)	12 (21.4)	2 (8.7)	10 (30.3)	
Duration of total TNFi exposure, mon (median, IQR)	27.8 (11.2–64.0)	42.8 (19.1–72.0)	24.1 (7.0–36.7)	**0.03**
Type of tuberculosis				
Lung limited, n (%)	22 (39.3)	11 (47.8)	11 (33.3)	0.28
Extrapulmonary, n (%)	14 (25.0)	6 (26.1)	14 (42.4)	0.21
Disseminated, n (%)	20 (35.7)	6 (26.1)	8 (24.2)	0.88
Increased AS activity after stopping TNFi, n (%)	36 (64.3)	21 (91.3)	15 (45.5)	**<0.001**

AS: ankylosing spondylitis; BMI: body mass index; LTBI: latent tuberculosis infection; TNFi: tumor necrosis factor inhibitor; INH: isoniazid; RIF: rifampin.

Among patients with active tuberculosis (n = 56), fourteen patients (25.0%) had extrapulmonary and twenty (35.7%) had disseminated tuberculosis. Interestingly, 2/3 of patients (67.9%) experienced tuberculosis infection after 1-yr exposure of TNF inhibitors and 1/4 of patients (26.8%) had been treated for LTBI before initiation of TNF inhibitor. These findings suggest the possibility of new tuberculosis outweighing reactivation of LTBI.

Anti-tuberculosis chemotherapy was started on the basis of clinical (n = 18), microbiological (n = 36) or pathological (n = 2) findings. Of these 56 AS patients, 23 resumed TNF inhibitors after initiating the anti-tuberculosis treatment (15 patients during anti-tuberculosis treatment and 8 after completion of anti-tuberculosis treatment).

### Resumers versus non-resumers

Baseline demographics of the AS patients who resumed TNF inhibitors or did not resume after active tuberculosis are compared in Table 1. TNF inhibitors were resumed a median of 5.8 months (0–15.8 months) after stopping them. Resumers had been treated with TNF inhibitors for longer than non-resumers (42.9 vs. 24.1 months, *P* = 0.03). Otherwise baseline features were not different between the groups, including LTBI and history of cured tuberculosis before anti-TNF treatment.

All resumers, except 1 who had stable disease and 1 with unavailable data, reported to have worsened AS disease activity while 15 patients among non-resumers had worsened disease activity, with 13 stable and 4 improved disease activity during suspension of TNF inhibitor. Resumers experienced disease flares from patients’ perspective more frequently than non-resumers (91.3% vs. 45.5%, *P* < 0.001). Non-resumers were treated more often with glucocorticosteroid, NSAIDs and DMARDs instead of TNF inhibitor. Paradoxical response after discontinuation of etanercept was reported from a patient in non-resumer group. Sudden onset of pleural effusion after 2 months of discontinuation of etanercept while receiving proper tuberculosis chemotherapy was noted in that patient, eventually, the diagnosis of paradoxical response following discontinuation of TNF inhibitor was made.

*Mycobacterium tuberculosis* cultures from sputum and/or tissue specimens were available in 26 patients, and were tested for sensitivity to first-line anti-tuberculosis drugs. Among these patients were four whose cultures showed some drug resistance and they were not restarted on TNF inhibitors.

### Choosing TNF inhibitors among resumers

As shown in Table 2, frequently recommenced TNF inhibitors were etanercept (n = 11), followed by adalimumab (n = 6), infliximab (n = 4). Among the 23 resumers, 10 were maintained on the same TNF inhibitor (adalimumab (n = 4), infliximab (n = 3), etanercept (n = 3)), while 13 were switched to another. Eight of the patients, who had received infliximab, were switched to etanercept (n = 4), adalimumab (n = 2), golimumab (n = 1) and certolizumab (n = 1). Four adalimumab users changed to etanercept and 1 etanercept user changed to infliximab. Descriptive clinical information on AS patients who resumed TNF inhibitors are given in Table 2.

**Table 2:** Clinical information on AS patients who resumed TNF inhibitors.

	Pt No.	Age(yr)	Sex	Disease duration (yr)	Time to tuberculosis from initiation of TNFi (mon)	LTBI treatment	TNFi before tuberculosis	Resumed TNFi	AS disease activity after TNFi suspension	Time to resuming TNFi (mon)	Follow-up duration since resuming TNFi (mon)
Early resumer	#1	61	M	6	4		ADA	ADA	Worsened	3	49
	#2	36	M	10	1	RFP	ETC, IFX	ADA	Worsened	3	67
	#3	43	M	7	39		ADA, ETC, IFX	CER	Worsened	5	10
	#4	43	M	10	16	INH	ETC, IFX	ADA	Worsened	5	21
	#5	56	M	12	25		ETC, ADA	ADA	Stable	4	37
	#6	44	M	8	5		IFX, ETC	ETC	Worsened	3	85
	#7	48	M	15	48	RFP	IFX	IFX	Worsened	3	4
	#8	60	M	9	20	INH	IFX	IFX	Worsened	2	67
	#9	25	M	7	72		ADA	ADA	Worsened	5	4
	#10	23	F	2	2		ETC	ETC	Unavailable	0	21
	#11	32	M	10	16		IFX	ETC	Worsened	6	79
	#12	51	M	17	99		IFX, ETC	IFX	Worsened	3	48
	#13	48	M	10	19		IFX	IFX	Worsened	4	88
	#14	44	M	8	36		ETC, IFX	ETC	Worsened	3	21
	#15	36	M	19	43		ADA	ETC	Worsened	4	21
Late resumer	#16	33	M	17	21		ADA	ETC	Worsened	7	80
	#17	28	M	7	68		IFX	ETC	Worsened	7	22
	#18	63	M	6	21	INH	ADA	ETC	Worsened	9	34
	#19	66	M	5	2		IFX	ETC	Worsened	16	33
	#20	51	M	11	59	INH	ETC	ETC	Worsened	8	35
	#21	40	M	9	23		GOL, IFX	GOL	Worsened	9	15
	#22	45	M	13	35		ADA	ADA	Worsened	13	17
	#23	33	M	17	12		ADA	ETC	Worsened	12	59

LTBI: latent tuberculosis infection; TNFi: tumor necrosis factor inhibitor; IFX: infliximab; ADA: adalimumab; GOL: golimumab; ETC: etanercept; CER: certolizumab

### Timing and safety of resuming TNF inhibitors

Eight patients (late resumers) restarted TNF inhibitor after completion of anti-tuberculosis treatment while 15 patients (early resumers) restarted during anti-tuberculosis treatment (median duration 9.0 (IQR 7.4–12.6) vs. 3.3 (2.9–4.7) months) (Table 3). Baseline features were not different between the early and late resumer groups, including LTBI and history of cured tuberculosis before anti-TNF treatment. Among the early resumers, one (#10) who had been exposed to etanercept for 2 months continued on it along with the anti-tuberculosis regimen. Despite recommencement of TNF inhibitor during or after tuberculosis treatment, the tuberculosis were treated successfully in all patients as scheduled, and did not recur during follow-up [median 33.8 (IQR 20.8–66.7) months; early resumers 37.0 (20.8–67.4) and late resumers 33.6 (18.5–52.7)]. Further adverse events after re-administration of TNF inhibitors were not reported during follow-up.

**Table 3:** Comparison of clinical features of early resumers and late resumers of TNF inhibitor in AS patients who experienced active tuberculosis infection.

	Early Resumer (n = 15)	Late-resumer (n = 8)	p-value
Age, yr (median, IQR)	44.0 (35.6–48.6)	36.0 (29.4–43.9)	0.25
Male sex, n (%)	14 (93.3)	8 (100.0)	0.46
Disease duration, yr (median, IQR)	9.3 (6.7–11.3)	9.5 (5.6–15.5)	0.76
BMI, kg/m^2^ (median, IQR)	24.0 (22.2–27.0)	24.0 (20.4–24.5)	0.83
LTBI, n (%)	4 (33.3)	2 (25.0)	0.69
Previous tuberculosis history, n (%)	3 (20.0)	2 (25.1)	0.78
Concomitant treatment			
Methotrexate, n (%)	5 (33.3)	2 (25.0)	0.68
Sulfasalazine, n (%)	1 (6.7)	1 (12.5)	0.63
Glucocorticosteroids, n (%)	2 (13.3)	2 (25.0)	0.48
Type of TNFi at tuberculosis			0.41
Infliximab, n (%)	10 (66.7)	3 (37.5)	
Adalimumab, n (%)	4 (26.7)	4 (50.0)	
Etanercept, n (%)	1 (6.7)	1 (12.5)	
Duration of total TNFi exposure, mon, median (IQR)	45.9 (19.1–79.0)	27.7 (13.9–65.0)	0.38
Type of tuberculosis			
Lung limited, n (%)	9 (60.0)	2 (25.0)	0.11
Extrapulmonary, n (%)	4 (26.7)	2 (25.0)	0.93
Disseminated, n (%)	2 (13.3)	4 (50.0)	0.06
Increased AS activity after stopping TNFi, n (%)	13 (86.7)	8 (100.0)	0.28
Type of TNFi when resuming			0.09
Infliximab, n (%)	4 (26.7)	0 (0.0)	
Adalimumab, n (%)	5 (33.3)	1 (12.5)	
Etanercept, n (%)	5 (33.3)	6 (75.0)	
Certolizumab, n (%)	0 (0.0)	1 (12.5)	
Golimumab, n (%)	1 (6.7)	0 (0.0)	
Time to resume after tuberculosis diagnosis, mon, median (IQR)	3.3 (2.9–4.7)	9.0 (7.4–12.6)	< 0.001
Follow up duration since restarting TNFi, mon, median (IQR)	37.0 (20.8–67.4)	33.6 (18.5–52.7)	0.71

AS: ankylosing spondylitis; BMI: body mass index; LTBI: latent tuberculosis infection; TNFi: tumor necrosis factor inhibitor.

## Discussion

Patients who develop active tuberculosis following TNF inhibitor use typically discontinue TNF inhibitor use as soon as possible, and reuse of TNF inhibitors has been opposed because of concerns about recurrence of tuberculosis. However, unlike the situation with RA, there are few options for controlling AS disease activity. Here, we found favorable long term outcomes of active tuberculosis in AS patients when they resumed TNF inhibitors. Furthermore, the outcomes were not different in patients who resumed TNF inhibitors before completion of anti-tuberculosis treatment or after completion.

There have been few studies of the outcome and safety of re-administration of TNF inhibitors in AS patients who were infected with tuberculosis due to their previous use [20–22]. One Korean report showed 3 patients resumed TNF inhibitors before the completion of tuberculosis treatment and 1 patient restarted TNF inhibitors one month after tuberculosis treatment completion and relapse did not occur in the mean 32 months of duration [20]. Suh *et al*. reported 10 cases of tuberculosis during TNF inhibitor treatment in 437 AS patients from a single center. Five patients among them restarted anti TNF treatment after a mean of 14 months of tuberculosis diagnosis without relapse within those months or the following period [21]. In France, 2 AS patients from a national registry who developed active tuberculosis while receiving infliximab recommenced TNF inhibitor without relapse, were reported by a postal questionnaire [22]. These observational studies have not detected any relapse of tuberculosis after TNF inhibitors recommencement in patients who had experienced TNF inhibitor-related tuberculosis and therefore, TNF inhibitors could be resumed in tuberculosis patients after appropriate anti-tuberculosis treatment. However, the studies that exist involved relatively small AS cohorts. In our study using a nationwide registry in an area with intermediate tuberculosis burden, 23 patients restarted TNF inhibitors after initiating anti-tuberculosis treatment without aggravation of the tuberculosis, delay of the treatment or tuberculosis-related deaths. Our results provide evidence and further extend the findings of these studies, indicating re-initiation is safe when supported by adequate anti-tuberculosis treatment.

Limitations of guidelines on continuation or recommencement of TNF inhibitor after tuberculosis infection stem from lack of published studies attempting to examine the issue. The guidelines of the British Thoracic Society address continuation of TNF inhibitor if clinically indicated and if patients are under appropriate tuberculosis treatment [5]. The belief that tuberculosis is aggravated by continued use of TNF inhibitor may have led to the almost universal recommendation to discontinue TNF inhibitor [10, 11]. Indeed, clinicians were concerned by the increased prevalence of tuberculosis in patients receiving TNF inhibitors, and particularly by the noticeable emergence of extra-pulmonary/disseminated and rapidly progressive tuberculosis [23]. However, a study from Portugal recently reported that the outcomes of 25 patients with inflammatory bowel disease who were infected by *Mycobacterium tuberculosis* while taking anti-TNF treatment were not worse than those of community-acquired tuberculosis, despite the higher frequency of the extra-pulmonary type in the TNF inhibitor-associated tuberculosis [24]. Additionally, tuberculosis-related deaths in AS patients are less frequent than in RA patients where comorbid conditions may play a role [8]. TNF inhibition can enhance anti-tuberculosis drug efficacy by disrupting granuloma, resultingly *Mycobacterium tuberculosis* transit from a dormant into a replicating state [25]. Moreover, cases with clinical and radiological deterioration during appropriate anti-tuberculosis treatment, termed paradoxical response, have been reported after stopping TNF inhibitor and successfully treated with re-addition of TNF inhibitor [26]. Both theoretical enhancement of anti-tuberculosis drug efficacy and the successful treatment of paradoxical worsening of tuberculosis by addition of TNF inhibitor offer additional grounds for early reusing TNF inhibitor after tuberculosis.

In our registry, the most frequently resumed TNF inhibitor was etanercept, a recombinant protein of soluble TNF receptor, which has been considered having lesser risk of tuberculosis compared to monoclonal antibodies [7]. In the same vein, experts recommend using other biologics with lesser risk of tuberculosis such as ustekinumab for psoriatic arthritis or rituximab for rheumatoid arthritis after tuberculosis treatment instead of TNF inhibitor reinitiating [17]. Non-TNF biologics such as ustekinumab and secukinumab can be alternatives to AS patients who experienced TNF inhibitor-associated tuberculosis, are currently being introduced as new biologics for AS. These two biologics risks for development of tuberculosis are yet to be determined [27]. Also, they are not approved for AS treatment in Korea to date. Therefore, given the limited option to control AS activity, present data are important to physicians as they support evidence for resuming TNF inhibitors including monoclonal antibodies in an awkward situation.

In our registry, two thirds of patients with active tuberculosis showed negative screening results for LTBI prior to starting TNF inhibitor and it took longer time (25.7 mon) to develop tuberculosis than previous reports in which presented active tuberculosis shortly after induction of TNF inhibitor consistent with reactivation of LTBI [6, 23]. There are several possible speculations. First, recent strategies to treat LTBI decrease tuberculosis reactivation associated with TNF inhibitor by 80% [28, 29], while long term use of TNF inhibitors drive recipients susceptible to new tuberculosis infection in the intermediate tuberculosis burden area. Second, the sensitivity of current LTBI screening methods could be suboptimal in immunocompromised hosts [30]. Thus, careful and continuous monitoring for development of tuberculosis is absolutely needed even for patients with a negative tuberculosis skin test or interferon gamma releasing assay result on initial evaluation.

Several limitations on the study could be addressed. First, the follow-up period after resuming TNF inhibitors may not have been enough. However, it was nearly as long as the exposure to TNF inhibitors leading up to the tuberculosis infection. Second, resuming protocols were not standardized. Physicians decided when to resume considering patients’ symptom and signs as well as their comorbidities. Accordingly, data on AS disease activity at the close time after TNF suspension were to track patient’s perspectives and classified with applying neither Bath Ankylosing Spondylitis Disease Activity Index nor Ankylosing Spondylitis Disease Activity Score. Third, patient information was collected in a retrospective manner. Nevertheless, the current study has strength in the data collected as it is from real practice in an intermediate tuberculosis burden area and involves larger numbers than those evaluated in previous reports. Moreover, the data composition is of multicenter registry covering patients treated in secondary or tertiary hospitals nationwide.

Ultimately, our nationwide study supports the view that TNF inhibitors can be resumed early in AS patients once active tuberculosis is under control. Sensitivity to the anti-tuberculosis drug should then be confirmed, and drug compliance and the formation of cavities, which are factors associated with treatment failure [31], should be carefully monitored during maintaining reintroduced TNF inhibitors.

## Supporting Information

S1 Table List of AS patients enrolled in current study. (XLSX)

S2 Table List of early resumers and late resumers. (XLSX)
